# Genome-wide association mapping for wheat blast resistance in CIMMYT’s international screening nurseries evaluated in Bolivia and Bangladesh

**DOI:** 10.1038/s41598-020-72735-8

**Published:** 2020-10-02

**Authors:** Philomin Juliana, Xinyao He, Muhammad R. Kabir, Krishna K. Roy, Md. Babul Anwar, Felix Marza, Jesse Poland, Sandesh Shrestha, Ravi P. Singh, Pawan K. Singh

**Affiliations:** 1 International Maize and Wheat Improvement Center (CIMMYT), Apdo. Postal 6-641, 06600 Mexico, DF Mexico; 2 Bangladesh Wheat and Maize Research Institute (BWMRI), Nashipur, Dinajpur Bangladesh; 3 Instituto Nacional de Innovación Agropecuaria y Forestal (INIAF), La Paz, Bolivia; 4 Department of Plant Pathology, Wheat Genetics Resource Center, Kansas State University, Manhattan, KS USA

**Keywords:** Plant breeding, Agricultural genetics, Genome-wide association studies, Genetic markers

## Abstract

Wheat blast caused by the fungus *Magnaporthe oryzae* pathotype *Triticum* (MoT) is an emerging threat to wheat production. To identify genomic regions associated with blast resistance against MoT isolates in Bolivia and Bangladesh, we performed a large genome-wide association mapping study using 8607 observations on 1106 lines from the International Maize and Wheat Improvement Centre’s International Bread Wheat Screening Nurseries (IBWSNs) and Semi-Arid Wheat Screening Nurseries (SAWSNs). We identified 36 significant markers on chromosomes 2AS, 3BL, 4AL and 7BL with consistent effects across panels or site-years, including 20 markers that were significant in all the 49 datasets and tagged the 2NS translocation from *Aegilops ventricosa*. The mean blast index of lines with and without the 2NS translocation was 2.7 ± 4.5 and 53.3 ± 15.9, respectively, that substantiates its strong effect on blast resistance. Furthermore, we fingerprinted a large panel of 4143 lines for the 2NS translocation that provided excellent insights into its frequency over years and indicated its presence in 94.1 and 93.7% of lines in the 2019 IBWSN and SAWSN, respectively. Overall, this study reinforces the effectiveness of the 2NS translocation for blast resistance and emphasizes the urgent need to identify novel non-2NS sources of blast resistance.

## Introduction

Wheat blast caused by the ascomycete fungus *Magnaporthe oryzae* pathotype *Triticum* Catt. (MoT) (anamorph *Pyricularia oryzae* Cavara)^1–3^ is a pernicious and persistent threat to global wheat production that can cause severe yield losses^4–8^. The disease predominantly affects wheat spikes that become fully or partially bleached and result in poor quality, small-sized shriveled grains with a reduced test weight^4,9,10^. Following its initial detection in 1985 in the Brazilian state of Paraná^11^, the disease has spread to the country’s key wheat producing areas and was contained to Brazil for almost a decade^12–15^. However, the disease expanded into Bolivia’s Department of Santa Cruz in 1996^16^, and further into Paraguay and Argentina in 2002 and 2007, respectively^17–19^.

In 2016, the first wheat blast outbreak in Asia was reported in Bangladesh, and population genomic analysis indicated the likely role of the South American lineage of the fungus behind the epidemic^8,20^. While wheat imported from Brazil was considered to be the likely source of the disease^21^, a combination of warm and humid conditions during the heading stage triggered the epidemic^22^, that is generally favored by high temperatures between 25 and 30 °C and an increased wetting period^23^. The blast outbreak in Bangladesh escalated concerns of spread into the neighboring South Asian regions with similar environmental conditions^5^, and about seven million hectares in India, Pakistan and Bangladesh were estimated to be vulnerable to the disease^24^. Besides the alarming risk of blast in South Asia, the states of Louisiana, Mississippi and Florida in the U.S. were identified to have a high vulnerability to blast outbreaks^25^, indicating that further expansion of the disease cannot be overlooked.

Management of wheat blast using fungicides is possible, but has been reported to be elusive, because of the inefficiency of fungicides in offering complete control under high disease pressure, the resistance in MoT populations to some classes of fungicides, and the high cost of the fungicides that cannot be afforded by resource-poor farmers^4,6,9,26–28^. While other potential blast control measures including altering the sowing time^5,29^, suspending the cultivation of wheat in disease-prone areas and declaring a ‘wheat holiday’ will help to mitigate the disease spread to some extent, the development and deployment of wheat varieties with genetic resistance to blast has been resorted to as the most sustainable and farmer-friendly approach^4,30,31^.

Genetic resistance to wheat blast at the seedling stage follows a gene-for-gene interaction model^32^ and five resistance genes namely *Rmg2, Rmg3, Rmg7, Rmg8*, and *RmgGR119* have been identified in wheat against the MoT pathotype^33–37^. However, the genes *Rmg 2, Rmg 3* and *Rmg 7* do not confer resistance against the recent MoT isolates, and only *Rmg8* and *RmgGR119* have been reported to be effective against a range of recent MoT isolates and are promising for spike resistance also^4,37^. Besides these genes, the 2NS translocation from *Aegilops ventricosa* has been identified to be a promising source of wheat spike blast resistance^38,39^ and a recent bi-parental mapping study showed stable and high effects of the translocation on wheat blast resistance, accounting for 22.4–50.1% of the phenotypic variation in different environments^40^. However, it is not entirely reliable because the resistance is incomplete and sometimes background-dependent^38,41^. Hence, there is an urgent need to identify new resistance genes and sources of resistance to combat the deadly blast pathogen^4,5,41^.

The quest for novel blast resistance genes has been done conventionally using the linkage mapping approach, but is limited by the high population development time and the ability to identify only few segregating alleles that differ between the parents for resistance^42,43^. A powerful alternate to linkage mapping studies are genome-wide association studies (GWAS), that depend on the linkage disequilibrium (LD) between the markers and the causal polymorphisms for identifying marker-trait associations, involve no population development time as they can be performed on existing diversity panels and provide better resolution compared to linkage mapping by harnessing all the recombination events that have occurred historically in the population^44–47^. However, GWAS for wheat blast are scanty^39^ and that served as the major impetus for this study focusing on using GWAS for identifying genomic regions associated with blast resistance in CIMMYT’s International Bread Wheat Screening Nurseries (IBWSNs) and Semi-Arid Wheat Screening Nurseries (SAWSNs).

The IBWSNs and SAWSNs targeted for the irrigated and drought-susceptible semi-arid environments, respectively^48,49^ are ideal for exploring novel genetic resistance to blast as they are elite lines from CIMMYT’s wheat breeding program that are distributed to several locations globally and serve as CIMMYT’s key international germplasm disseminating vehicles^48^. A large-dataset of 8607 blast observations obtained by evaluating 1106 lines in two IBWSNs (50 and 51 IBWSN) and two SAWSNs (35 SAWSN and 36 SAWSN) at three wheat blast hotspots including Jashore, Bangladesh (23° 10′ N 89° 10′ E), Quirusillas, Bolivia (18° 20′ S 63° 57′ W) and Okinawa, Bolivia (17° 13′ S 62° 53′ W) were used for the dual purposes of identifying novel sources and genomic regions associated with blast resistance in the CIMMYT germplasm. Furthermore, our additional objectives were to: (i) perform a genomic fingerprinting of alleles at consistent markers associated with blast resistance across nurseries and environments (ii) estimate the frequencies of favorable alleles (FAs) for consistent blast associated markers and (iii) estimate the frequencies of the 2NS translocation in a large panel of 4143 lines comprising all the IBWSNs and SAWSNs distributed internationally between 2012 and 2019.

## Results

### Blast phenotyping data distributions and correlations

The datasets used in this study include lines from the following nurseries that were evaluated in two planting dates (first planting, FP and second planting, SP): (i) the 50 IBWSN and 35 SAWSN that were evaluated in Okinawa and Quirusillas during the 2018 cycle and in Jashore and Quirusillas during the 2019 cycle and (ii) the 51 IBWSN and 36 SAWSN that were evaluated in Jashore, Okinawa and Quirusillas during the 2019 cycle and in Quirusillas during the 2020 cycle (Supplementary Table S1). The distributions of blast indices indicated that on average 73.9 ± 13.3% of the lines across all the nurseries had blast indices of 0 and were highly resistant (Supplementary Table S2). While the Quirusillas 2018 SP 50 IBWSN dataset had the highest number of lines (91.1%) with a blast index of 0, the Jashore 2019 FP 35 SAWSN dataset had the least number of such lines (44.9%) (Supplementary Fig. S1). Overall, across all the panels, 83.7% of lines had a mean blast index less than 10, among which 27.4% of lines had a mean blast index of zero (Supplementary Fig. S1).

We also observed that the Okinawa 2019 environment had the highest mean blast index (12.8 ± 3.14), followed by Jashore 2019 (12.6 ± 3.5), Quirusillas 2019 (8.03 ± 1.8), Quirusillas 2018 (6 ± 1.1), Okinawa 2018 (4.8 ± 1.6) and Quirusillas 2020 (4.6 ± 0.9) environments. Considering the four nurseries, the 36 SAWSN had the highest mean blast index (10.1 ± 4), followed by the 35 SAWSN (9.2 ± 4.9), 51 IBWSN (8.5 ± 3.8) and the 50 IBWSN (6.9 ± 4.1) (Supplementary Table S2). The blast correlations across the different sites were analyzed (Supplementary Table S3) and we observed that Quirusillas and Okinawa had the highest mean correlations (0.78 ± 0.07), followed by Quirusillas and Jashore (0.61 ± 0.13) and Okinawa and Jashore (0.61 ± 0.12). Within sites, the average correlation across all the planting environments and years of evaluations were high in Okinawa (0.82 ± 0.09) and Quirusillas (0.78 ± 0.06), but moderate in Jashore (0.57 ± 0.1). There were no significant differences between the blast indices in the two plantings (at a p-value threshold of 0.001), except in the Jashore 2019 environment (p-value of 9.98E−6). The average narrow-sense heritability across the two planting times in the different environments was 0.72 ± 0.12, and it ranged between 0.49 and 0.87 (Supplementary Table S3). We also observed low average correlations of the blast indices with days to heading (0.1 ± 0.12, ranged between − 0.12 and 0.27) and height (0.01 ± 0.13, ranged between − 0.35 and 0.35) (Supplementary Table S4).

### Population structure analysis

**Figure 1:**
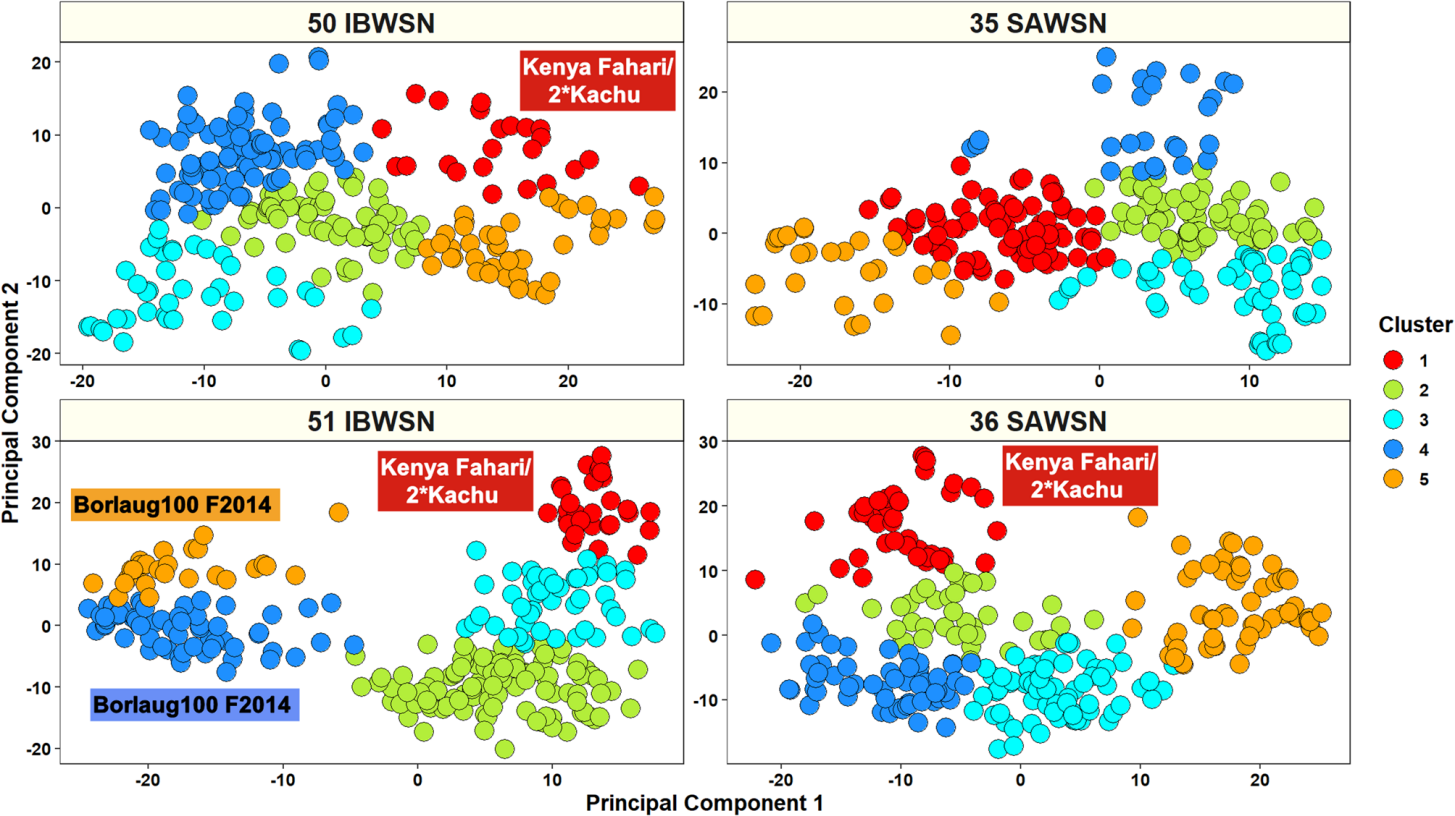
Population structure analysis in the 50 International Bread Wheat Screening Nursery (IBWSN) with 269 lines, 35 Semi-Arid Wheat Screening Nursery (SAWSN) with 265 lines, 51 IBWSN with 285 lines and 36 SAWSN with 287 lines.

Moderate population structure was observed in all the nurseries using the first two principal components (Fig. 1). The percentage variation explained by the first two principal components were: 8.9% and 5.4% in the 50 IBWSN, 7% and 4.3% in the 35 SAWSN, 13.7% and 8.8% in the 51 IBWSN and 10.7% and 7.9% in the 36 SAWSN, respectively. The principal components were also used to partition the lines into clusters using the ‘k-means’ clustering approach (Supplementary Table S5). In the 50 IBWSN, 78.3% of the lines in cluster 1 had the stem rust resistant parent Kenya Fahari/2*Kachu in the pedigree. In the 51 IBWSN, clusters 4 and 5 that clearly formed a separate group had the high yielding parent Borlaug100 F2014 in 92% of the lines, while cluster 1 had the parent Kenya Fahari/2*Kachu in 76% of the lines. In the 36 SAWSN, 60% of the lines in cluster 1 had parent Kenya Fahari/2*Kachu and 94.7% of the lines in cluster 5 had parent Borlaug100 F2014. In addition, we also observed that there were 9, 6, 11 and 8 ancestral sub-populations in the 50 IBWSN, 51 IBWSN, 35 SAWSN and 36 SAWSN, respectively (Supplementary Fig. S2).

### Genome-wide association mapping for blast resistance

**Figure 2:**
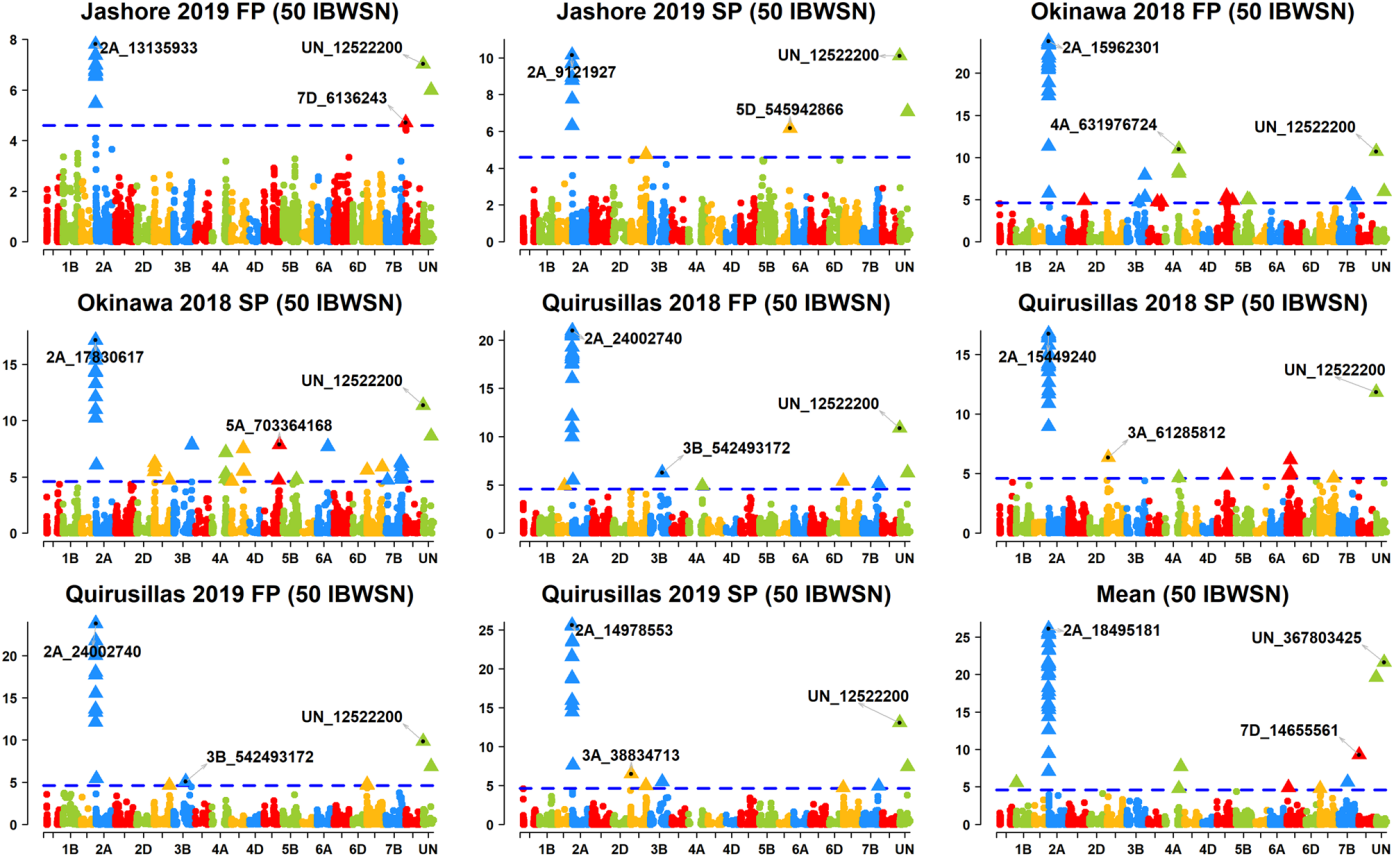
Markers significantly associated with blast resistance in the 50 International Bread Wheat Screening Nursery (IBWSN). A Bonferroni α level of 0.20 was used to correct for multiple testing and the most significant marker in each chromosome is indicated.

**Figure 3:**
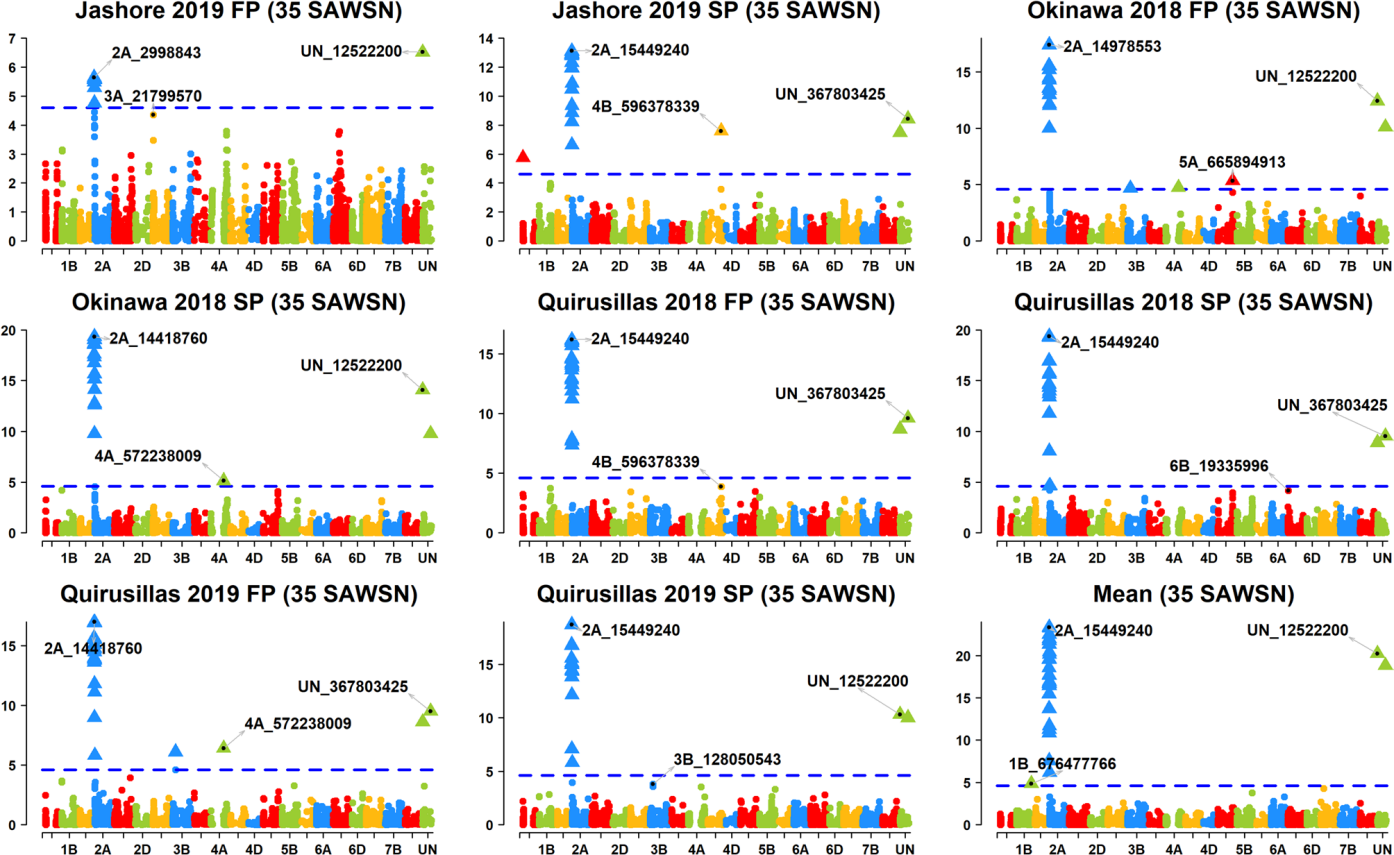
Markers significantly associated with blast resistance in the 35 Semi-Arid Wheat Screening Nursery (SAWSN). A Bonferroni α level of 0.20 was used to correct for multiple testing and the most significant marker in each chromosome is indicated.

**Figure 4:**
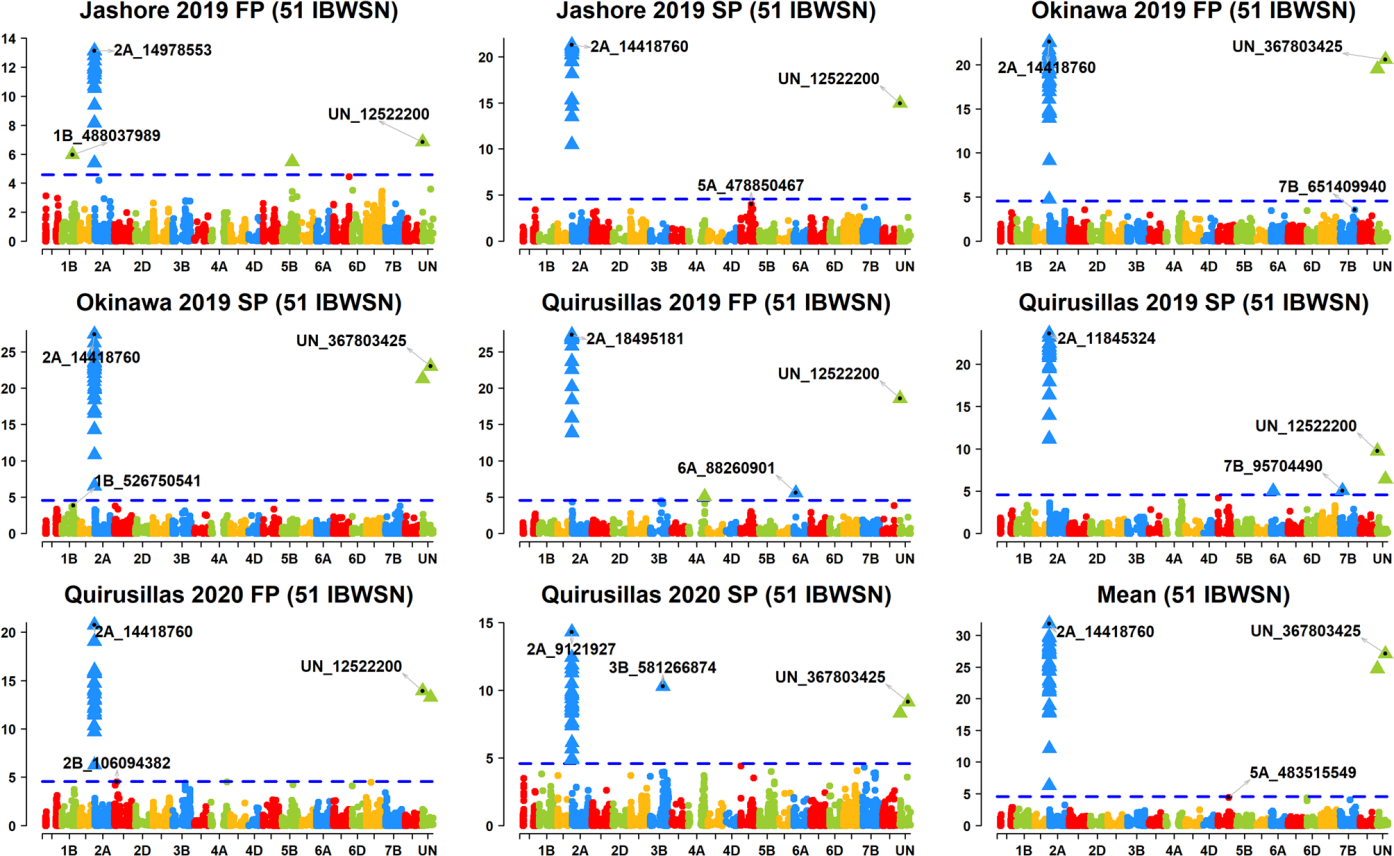
Markers significantly associated with blast resistance in the 51 International Bread Wheat Screening Nursery (IBWSN). A Bonferroni α level of 0.20 was used to correct for multiple testing and the most significant marker in each chromosome is indicated.

**Figure 5:**
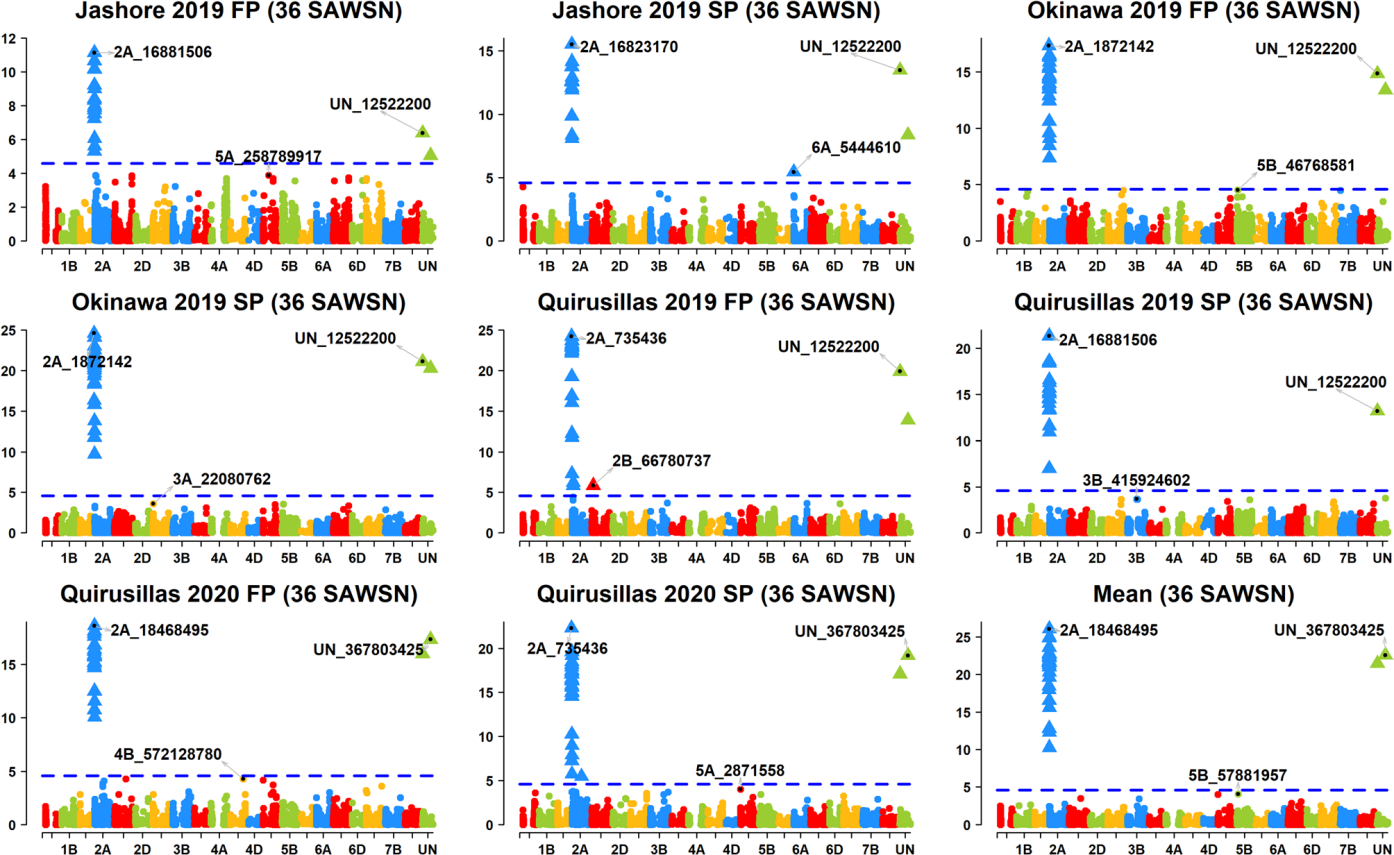
Markers significantly associated with blast resistance in the 36 Semi-Arid Wheat Screening Nursery (SAWSN). A Bonferroni α level of 0.20 was used to correct for multiple testing and the most significant marker in each chromosome is indicated.

**Figure 6:**
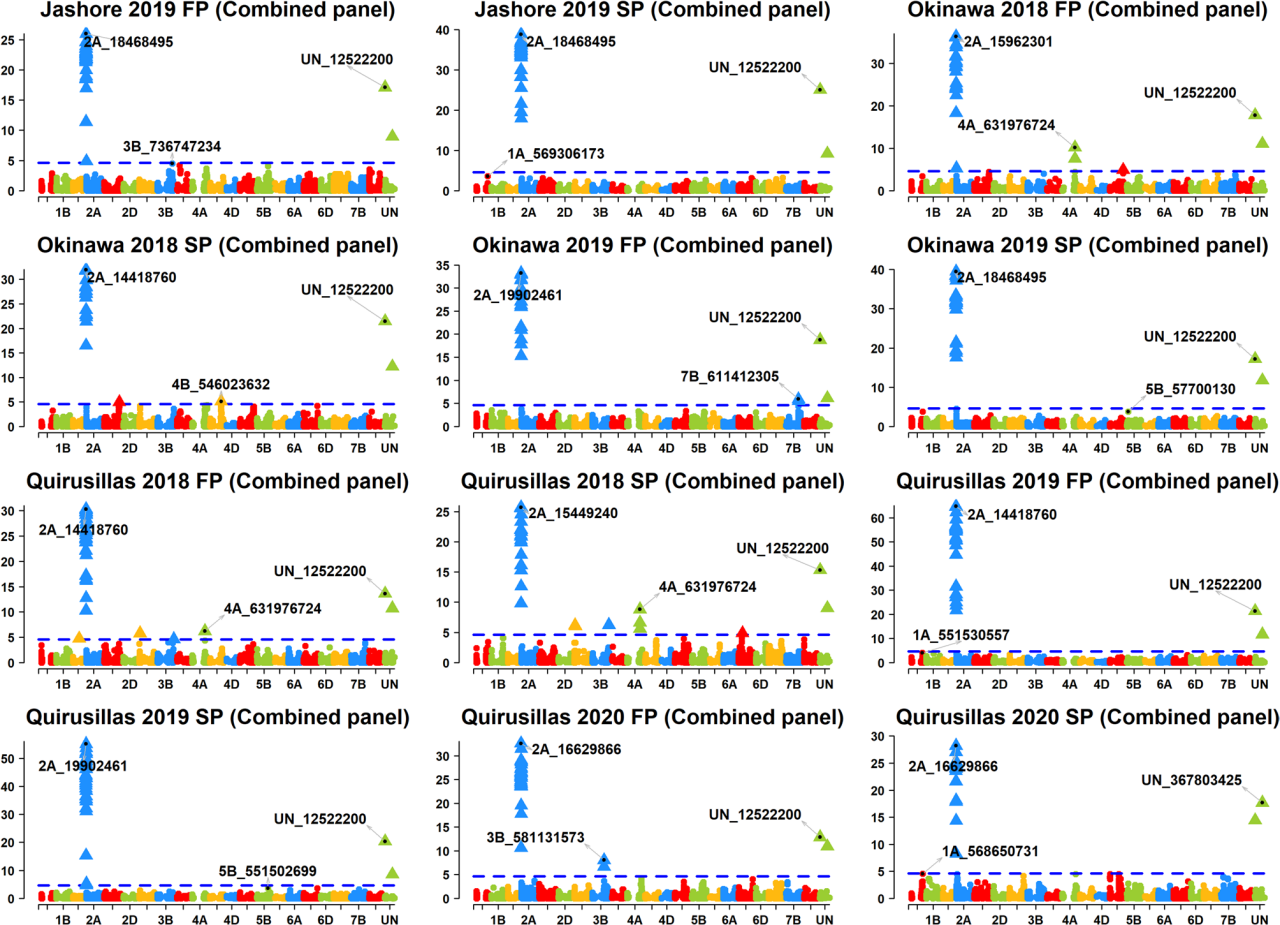
Markers significantly associated with blast resistance in the combined panel comprising the 50 and 51 International Bread Wheat Screening nurseries, 35 and 36 Semi-Arid Wheat Screening Nurseries. A Bonferroni α level of 0.20 was used to correct for multiple testing and the most significant marker in each chromosome is indicated.

Across the 49 datasets, we identified 114 markers significantly associated with blast resistance after Bonferroni correction for multiple testing (α level of 0.20, Figs. 2, 3, 4, 5, 6, Supplementary Fig. S1) and have reported the marker p-values, additive effects (AEs) and the percentage variation explained by them (Supplementary Table S6). While these markers were present in all chromosomes except 2D and 4D, the chromosomes that had the highest number of markers were 2A (41), 3B (26), 5A (9), 7B (6), 4A (6) and 5B (5). However, only 74 markers on chromosomes 2A, 3A, 3B, 4A, 5A, 5B, 6A, 7A and 7B were significant in more than one dataset, with the highest number of markers on chromosomes 2A (31), 3B (17), 5A (7) and 4A (5).

### Markers associated with blast resistance in the 50 IBWSN

In the 50 IBWSN, the most significant markers in the nine datasets were on chromosome 2AS and included markers 2A_13135933, 2A_9121927, 2A_15962301, 2A_17830617, 2A_24002740, 2A_15449240, 2A_14978553 and 2A_18495181 (Fig. 2). Across the different environments, we identified 62 repeatable markers, among which 51 markers on chromosomes 2AS, 3AL, 3BL, 4AL, 5AL, 5BL and 7BL had average AEs greater than 1. On chromosome 2AS, 25 markers between 718,152 and 24,002,740 bps (0–8.9 cM) were significant in all the datasets with an average AE of 22.3 ± 5.8 on the blast index and explained on average 37.2 ± 11.5% of the blast variation. In addition, marker 2A_52273147 (46.8 cM) also on chromosome 2AS was significant in the Okinawa 2018 SP, Quirusillas 2018 FP, Quirusillas 2019 FP and SP environments with an average AE of 3.4 ± 1.5. On chromosome 3AL, marker 3A_708005281 (141.2 cM) was significant in the Quirusillas 2019 FP and SP environments with an average AE of 12.4 ± 0.4. On chromosome 3BL, marker 3B_542493172 (75.6 cM) was significant in the Okinawa 2018 FP, Quirusillas 2018 FP and Quirusillas 2019 FP and SP environments with an average AE of 9.7 ± 1.9. In addition, 11 markers on chromosome 3BL between 823,763,468 and 829,382,536 bps (161.3 cM) were significantly associated in the Okinawa 2018 FP and SP environments but had a low average AE of 1.2 ± 0.8. On chromosome 4AL, markers 4A_631976724, 4A_631976793 and 4A_636046879 (79.6 cM) were significantly associated in the Okinawa 2018 FP and SP environments with a low average AE of 1.2 ± 0.3. On chromosome 5BL, four markers 5B_616367516, 5B_616955782, 5B_617620584 and 5B_618146508 (134.2 cM) were significantly associated in the Okinawa 2018 FP and SP environments with a low average AE of 1.1 ± 0.4. On chromosome 7BL, marker 7B_730272557 (169.5 cM) was significantly associated with the Quirusillas 2018 FP and 2019 SP datasets with an average AE of 2.4 ± 0.3.

### Markers associated with blast resistance in the 35 SAWSN

In the 35 SAWSN, marker 2A_15449240 on chromosome 2AS was the most significant marker in five datasets including Jashore 2019 SP, Quirusillas 2018 FP and SP, Quirusillas 2019 SP and the mean (Fig. 3). In the Jashore 2019 FP, Okinawa 2018 FP and SP and Quirusillas 2019 FP datasets, the most significant markers included UN_12522200, 2A_14978553, 2A_14418760 and 2A_14418760, respectively. Across the different environments, 34 repeatable markers were identified on chromosomes 2AS, 3BS and 4AL. On chromosome 2AS, 28 markers between 718,152 and 24,002,740 bps were significant in eight to nine datasets with an average AE of 17.5 ± 4.3 and explained on average 30.5 ± 9.2% of the blast variation. On chromosome 3BS, marker 3B_128050543 (59.7 cM) was significantly associated in the Quirusillas 2019 FP and Okinawa 2018 FP environments with an average AE of 2 ± 1.7. On chromosome 4A, markers 4A_572238009 and 4A_572238015 at 29 cM were significantly associated in the Quirusillas 2019 FP, Okinawa 2018 FP and SP datasets with an average AE of 2.4 ± 0.6.

### Markers associated with blast resistance in the 51 IBWSN

In the 51 IBWSN, marker 2A_14418760 on chromosome 2AS was the most significant marker in Jashore 2019 SP, Okinawa 2019 FP and SP, Quirusillas 2020 FP and the mean. Markers 2A_14978553, 2A_18468495, 2A_11845324 and 2A_9121927 were the most significant in the Jashore 2019 FP, Quirusillas 2019 FP and SP and in the Quirusillas 2020 SP datasets, respectively (Fig. 4). We identified 31 repeatable markers on chromosomes 2AS and 6AS that were significantly associated across the different datasets. Among them, 28 markers between 718,152 and 24,002,740 bps on chromosome 2AS were significantly associated in all the datasets with an average AE of 24 ± 8.6 and explained on average 36.8 ± 14.8% of the variation. On chromosome 6AS, marker 6A_88260901 (53.8 cM) was significantly associated in the Quirusillas 2019 FP and SP datasets with an average AE of 2.4 ± 1.5.

### Markers associated with blast resistance in the 36 SAWSN

In the 36 SAWSN, markers 2A_16823170, 2A_16881506, 2A_18468495, 2A_1872142 and 2A_735436 on chromosome 2AS were the most significant markers (Fig. 5). Across the different datasets, 30 markers were significantly associated with blast resistance, including 28 markers on chromosome 2AS between 718,152 and 24,002,740 bps that were significant in all the datasets with an average AE of 21.1 ± 6.3 and explained on average 31.4 ± 11.4% of the blast variation.

### Markers associated with blast resistance in the combined panel

In the combined panel, the most significant markers in the different datasets included markers 2A_14418760, 2A_15449240, 2A_15962301, 2A_16629866, 2A_18468495, 2A_18495181 and 2A_19902461 on chromosome 2AS (Fig. 6, Supplementary Fig. S3). Across the different environments, we identified 37 repeatable on chromosomes 2AS, 3AS, 3BL and 4AL. On chromosome 2AS, 28 markers between 718,152 and 24,002,740 bps were significant in all the datasets with an average AE of 19.9 ± 6.2 and explained on average 23.8 ± 7.3% of the blast variation. On chromosome 3AS, marker 3A_38834713 (35 cM) was significant in the Quirusillas FP and SP datasets with an average AE of 1.2 ± 1.6. On chromosome 3BL, three markers 3B_823875455, 3B_823964519 and 3B_823984989 (161.3 cM) were significantly associated in the Quirusillas 2018 FP and SP datasets with average AE of 0.97 ± 0.3. On chromosome 4AL, markers 4A_631976724 and 4A_631976793 (79.6 cM) were significantly associated in the Okinawa 2018 FP, Quirusillas 2018 FP and SP, and in the mean dataset, but had a very low average AE of 0.31 ± 0.18.

### Markers associated with blast resistance across nurseries and sites

Across the four nurseries and three sites, 40 markers were significantly associated with blast resistance which included: 20 markers on chromosome 2AS and marker UN_12522200 significant in all the 49 datasets, six markers on chromosome 2AS significant in 48 datasets, marker UN_367803425 significant in 43 datasets, marker 2A_16881506 significant in 42 datasets and marker 2A_19914469 significant in 39 datasets. Besides these markers, we observed that marker 2A_52273147 was significant in four datasets in the 50 IBWSN and one in the 35 SAWSN. The other markers that were significant across different sites include, (i) 4A_631976724 significant in seven datasets in Okinawa and Quirusillas 2018 (ii) 2A_52273147 significant in five datasets in Okinawa 2018, Quirusillas 2018 and 2019 (iii) 4A_631976793 and 4A_636046879 significant in four datasets in Okinawa and Quirusillas 2018 (iv) 3B_542493172 significant in four datasets in Okinawa 2018, Quirusillas 2018 and 2019 (v) 4A_572238009 and 4A_572238015 significant in three datasets in Okinawa 2018 and Quirusillas 2019 (vi) 3B_128050543 significant in Okinawa 2018 FP and Quirusillas 2019 FP and (vii) 2A_28784613 significant in Jashore 2019 FP and Okinawa 2018 FP.

### Genomic fingerprinting of lines for markers consistently associated with blast resistance

**Figure 7:**
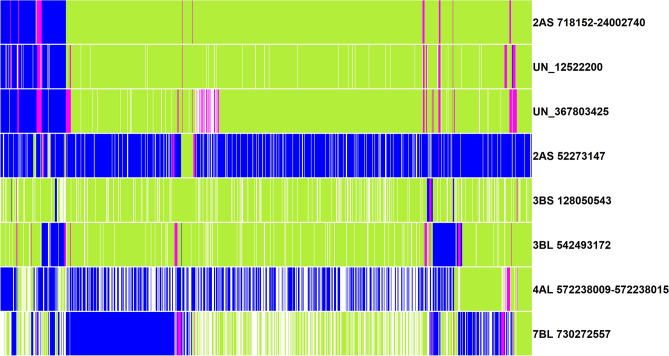
Genomic fingerprinting of blast associated markers and regions in 1106 lines from the the 50 and 51 International Bread Wheat Screening nurseries and 35 and 36 Semi-Arid Wheat Screening Nurseries. The green color indicates the favorable allele (allele with a decreasing effect on the blast index), the blue color indicates the unfavorable allele (allele with an increasing effect on the blast index), the magenta color indicates the heterozygote and the white color indicates missing data.

Among the markers that were significant in more than one panel or site-year, we observed that only 36 had consistent allelic effects across the datasets. This included 28 markers on chromosome 2AS between 718,152 and 24,002,740 bps, in addition to markers 2A_52273147, 3B_128050543, 3B_542493172, 4A_572238009, 4A_572238015, 7B_730272557, UN_12522200 and UN_367803425. Based on the allelic effects, the alleles of these markers were designated as the FA, unfavorable allele and the heterozygote and used to fingerprint 1106 lines in all the nurseries (Fig. 7, Supplementary Table S7). The markers on chromosome 2AS between 718,152 and 24,002,740 bps and the markers 4A_572238009 and 4A_572238015 on chromosome 4AL had high LD between them and hence the most consistent allele at those regions were obtained. We observed that the FAs for marker 3B_128050543 were present in the highest number of lines (97.5%), followed by marker 3B_542493172 (88%), the 2AS 718,152–24,002,740 region (87.6%), markers UN_12522200 (86.1%), UN_367803425 (81.4%), 7B_730272557 (46.4%) and the 4AL 572,238,009–572,238,015 region (25.1%).

**Figure 8:**
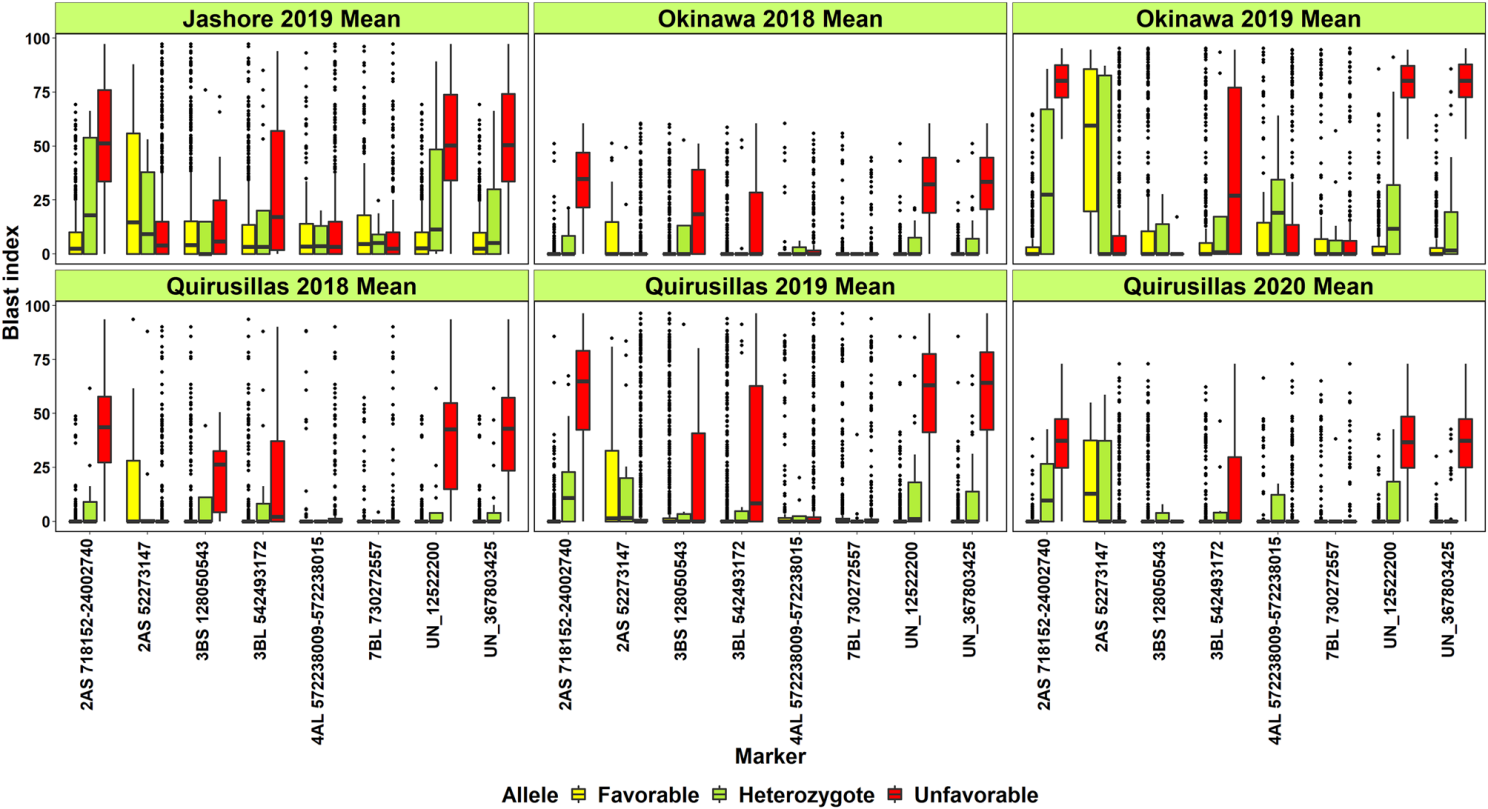
Box plots of marker alleles in 1106 lines plotted against the mean blast indices of the lines evaluated in Jashore 2019, Okinawa 2018 and 2019, Quirusillas 2018, 2019 and 2020. The favorable allele is the allele with a decreasing effect on the blast index and the unfavorable allele is the allele with an increasing effect on the blast index.

Considering the allelic effects at the 36 consistent markers and the mean blast indices of the 1,106 lines evaluated in the six environments (Fig. 8), the 2AS 718,152–24,002,740 bps region, markers UN_12522200 and UN_367803425 had large differences in the blast indices for the FA and the unfavorable allele across all the environments. In addition, markers 3B_128050543 and 3B_542493172 also had moderate differences in several environments, but none of the other markers showed consistent allelic effects across the different environments. We then compared the consensus FA at the markers on chromosome 2AS between 718,152 and 24,002,740 bps to the presence or absence of the 2NS translocation scored using the Kompetitive allele specific polymerase chain reaction marker CIMwMAS0004^50–52^. The coincidence of the FAs in 98.8% of the lines (excluding the heterozygotes) with the presence of the 2NS translocation indicated clearly that the most consistent markers in this study are in the 2NS translocation (Supplementary Table S7).

To understand if the presence of FAs for markers other than the 2NS translocation in the lines with the 2NS translocation had an effect on the blast severity in each of the environments, we fitted a linear regression model with the blast severity as the response and the number of FAs at markers 2AS 52,273,147, 3B_128050543, 3B_542493172, 7BL 730,272,557 and the 3BL 823,763,468–824,374,886 and 4AL 572,238,009–572,238,015 regions as the independent variable and observed that they did not have a significant effect on the blast severity in any of the environments, with the p-values ranging between 0.1 and 0.98. Similarly, we also fitted another linear regression model with the number of FAs at the aforementioned markers vs the response variable which was the mean blast severity of all the lines and observed a significant effect in Okinawa 2018 FP, Okinawa 2018 SP, Quirusillas 2019 SP, Quirusillas 2018 FP and the mean datasets, but the effect size on the blast index was very small and between 2.8 and 4.3.

### Blast indices in the lines with and without the 2NS translocation

We observed that 95.03% of the lines with the 2NS translocation had mean blast indices less than 10, 31.3% of the lines had blast indices of zero, 4.6% of the lines had mean blast indices between 10 and 30 and three lines had blast indices between 32.2 and 45.6. The mean blast indices in the lines with and without the 2NS translocation were 2.7 ± 4.5 and 53.3 ± 15.9, respectively. Among the 17 heterozygotes, the mean blast indices were zero in five lines, ranged between 0.2 and 18.7 in five lines and between 23.13 and 63.6 in seven lines. We also analyzed the progenies in 36 crosses with the 2NS translocation and more than five full-sibs per cross and observed that two families had a range greater than 20 in the blast indices including: (i) BECARD/AKURI/3/KINGBIRD #1//INQALAB 91*2/TUKURU/4/BECARD/AKURI with mean blast indices ranging between 0 and 32.2 (ii) KACHU/BECARD//WBLL1*2/BRAMBLING/3/ATTILA*2/PBW65//MURGA with mean blast indices ranging between 0.5 and 22.4. We then clustered the 915 lines with the 2NS translocation based on the alleles at all the 28 markers in the translocation (Supplementary Fig. S4). We observed that 761 of the 915 lines had FAs at all the non-missing markers in the translocation, but their blast indices ranged between 0 and 45.6. Furthermore, we also visualized the blast indices, FAs and the unfavorable alleles at the 28 markers tagging the 2NS translocation in a subset of 65 lines that had consensus 2NS FAs, but also had some unfavorable alleles and heterozygotes (Supplementary Fig. S5). While the blast indices of these lines ranged between 0 and 29.2, there was no clear relationship between the presence of few unfavorable alleles or the heterozygote and the blast index.

Considering the 117 lines without FAs at the 2NS translocation, 62.5% of them had mean blast indices greater than 50, 31.3% of them had mean blast indices between 30 and 50, and seven lines had mean blast indices less than 30 (Supplementary Table S8), among which only one UP2338*2/4/SNI/TRAP#1/3/KAUZ*2/TRAP//KAUZ/5/MILAN/KAUZ//CHIL/CHUM18/6/UP2338*2/4/SNI/TRAP#1/3/KAUZ*2/TRAP//KAUZ/7/ATTILA*2/PBW65*2//KACHU/8/UP2338*2/4/SNI/TRAP#1/3/KAUZ*2/TRAP//KAUZ/5/2*BAJ #1 (GID7397442 in the 35 SAWSN) had a mean blast index of zero. However, when this line was further analyzed for the presence of the 2NS translocation using two codominant sequence-tagged-site markers, WGGB156 and WGGB159^40^, they suggested the presence of the translocation. Among the remaining lines, NELOKI*2/4/SOKOLL//PBW343*2/KUKUNA/3/ATTILA/PASTOR (GID7402225 in the 50 IBWSN) was the only line where the absence of the 2NS translocation was confirmed with all the markers and also had a low mean blast index of 15.

### Frequencies of lines with the 2NS translocation in the IBWSNs and SAWSNs

To understand the frequencies of lines with the 2NS translocation, we obtained their frequencies in eight IBWSNs (45–52 IBWSN) and SAWSNs (30–37 SAWSN) distributed internationally between 2012 and 2019 and comprising 4143 lines (Supplementary Table S9). We observed that in the IBWSNs, the percentage of lines with the 2NS translocation had increased 113.8% in seven years, from 44% in 2012 to 94.1% in 2019. Similarly, in the SAWSNs, the percentage of lines with the 2NS translocation had increased 524.3%, from 15% in 2012 to 93.7% in 2019 (Supplementary Fig. S6).

## Discussion

In this study, 1,106 lines in the IBWSNs and SAWSNs were evaluated for wheat blast in Bolivia and Bangladesh, and we observed a high level of resistance, with 83.7% of them having mean blast indices less than 10. In the environments considered in this study, the planting time did not significantly affect the disease indices except in one dataset, in contrast to a previous observation^29^. In addition, traits like days to heading and height had low correlations with disease index which can be partly attributed to the low variability in the blast indices. The high correlations between blast indices in Bangladesh and Bolivia (mean of 0.61) is encouraging, indicating similarities in blast responses across these environments.

Multi-environment GWAS for blast resistance were performed providing valuable insights into the genetic architecture of resistance against different isolates of MoT in Bolivia and Bangladesh. The markers that were significantly associated with blast resistance in all the 49 datasets used were on the 2NS translocation and explained up to 71.8% of the phenotypic variation, clearly indicating the large and consistent effect of this region. The low mean blast indices (less than 10) in 95.03% of lines with the 2NS translocation and the high mean blast indices (greater than 30) in 93.8% of the lines without the 2NS translocation, in addition to the large difference in the mean blast index of lines with and without the 2NS translocation provide strong evidence to the high and consistent effect of the 2NS translocation on blast resistance^38,40^. In addition, we observed that a small percentage of lines (1.6%) were heterozygous at all or several markers at the 2NS translocation, implying that heterozygosity in this translocation is possible, despite reports that the 2A and the 2N chromosomes do not recombine^50^. However, we observed that only 31.3% of the lines with the 2NS translocation had a mean blast index of zero across all the environments, and the remaining lines had mean blast indices ranging up to 45.6. While this affirms the background-dependence and partial nature of the resistance^38^, we have also reported families with the 2NS translocation that clearly had a range in their mean blast indices, indicating the incomplete effect of the translocation in some families and the probable involvement of other genes with small effects additively contributing to high levels of resistance.

Besides the 2NS translocation and the two unaligned markers (UN_12522200 and UN_367803425) that had a high LD with the 2NS translocation and probably were in the same region, we observed only six markers on chromosomes 2AS, 3BL, 4AL and 7BL that were significant in more than one nursery or environment. However, none of them had significant effects across all the six environments in this study, besides marker 3B_542493172 that was 7 cM away from marker 3B_476296464 reported to be associated with blast^39^. In addition, the combined presence of FAs for all the six markers had a low effect on the blast indices in few datasets indicating that the 2NS translocation is the only consistent region associated with blast resistance. The paucity of non-2NS based blast resistance sources is clearly demonstrated in our study by that only one line without the 2NS translocation had mean blast index of zero across all the environments. This can be attributed to the fact that only 10.7% of the lines used in this study lacked the 2NS translocation, among which 93.8% had mean blast indices greater than 30 and were susceptible.

We also fingerprinted a large panel of 4143 lines for the 2NS translocation that provided excellent insights into its frequency over years and indicated its presence in 94.1% and 93.7% of lines in the most recent IBWSN and SAWSN, respectively. This clearly indicates that future identification of novel non-2NS based resistance in the elite germplasm from CIMMYT might be challenging and evaluation of other genetic resources including synthetics, land races and exotic germplasm is essential to identify new sources of wheat blast resistant lines and genes. The remarkable increase in the proportion of lines with the 2NS translocation in the IBWSNs and SAWSNs (113.8 and 524.3%, respectively) over seven years is due to the association of the translocation with stripe rust (caused by *Puccinia striiformis*) resistance to races in Mexico and high grain yield^39^. However, the 2NS translocation has also been reported to be associated with lodging tolerance and resistance to stem rust caused by *Puccinia graminis*, leaf rust caused by *Puccinia triticina*, eyespot caused by *Pseudocercosporella herpotrichoides*, cereal cyst caused by *Heterodera avenae*, root knot caused by *Meloidogyne* spp.^39,53–57^ making it a very valuable region in wheat breeding.

While ‘Milan’ was the original source of the 2NS translocation in the CIMMYT germplasm, it has increased in frequency through ‘Milan’ derived lines like Mutus (MILAN/S87230/4/BOW/NAC//VEE/3/BJY/COC) and Kachu (KAUZ//ALTAR 84/AOS/3/MILAN/KAUZ/4/VEE/KOEL) that were used also widely as parents. The presence of the 2NS translocation in a large number of recent IBWSNs and SAWSNs is promising for short-term blast management and blast resistant varieties like ‘BARI Gom 33′ with the 2NS translocation have been released^58^. However, the reliance on only one resistance locus with a large effect is not recommended as it leads to selection pressure on the MoT populations^4,59^ and there are reports that the MoT isolates in Brazil^21^ and Paraguay^60^ have overcome the 2NS resistance. Hence, we would like to emphasize the importance of identifying other minor genes that can additively result in enhanced blast resistance and this strategy has been successfully used in breeding for durable resistance in other pathosystems such as the wheat-rust, where frequent breakdown of major genes occurs^61^. We also conclude that further research on identifying novel non-2NS sources of blast resistance is critical for diversifying and sustaining genetic resistance to wheat blast.

## Methods

### Nurseries and blast evaluations

In this study, we used 1,106 advanced CIMMYT wheat breeding lines from four nurseries: (i) 50 IBWSN with 269 lines (ii) 35 SAWSN with 265 lines (iii) 51 IBWSN with 285 lines and (iv) 36 SAWSN with 287 lines. The 50 IBWSN and 35 SAWSN were evaluated in Quirusillas during the 2018 and 2019 cycles, in Jashore during the 2019 cycle and in Okinawa during the 2018 cycle, while the 51 IBWSN and 36 SAWSN were evaluated in Jashore, Okinawa and Quirusillas during the 2019 cycle and in Quirusillas during the 2020 cycle. In the Department of Santa Cruz, Quirusillas, Bolivia, the lines were sown during the fourth week of December 2017, 2018 and 2019 in 1-m long double rows with 20-cm between-row spacing in two planting dates with the second planting done within 2 weeks after the first planting. In Okinawa, sowing was done in the second week of May 2018 and May 2019 with the same experimental design. Field inoculation in the two locations of Bolivia was done twice, with the first at anthesis and the second at two days after anthesis, with a backpack sprayer. Inoculum composed of five locally collected MoT isolates with high aggressiveness, QUI1505, QUI1601, QUI1612, OKI1503 and OKI1704, at a concentration of approximately 80,000 spores ml^−1^ of water^40^. Two local checks Urubo (resistant check) and Atlax (susceptible check) were used in the experiments in Bolivia.

In Jashore, Bangladesh, the nurseries were sown in late December 2018, with the same experimental design as in Bolivia. The inoculum was a mixture of six local MoT isolates, BHO17001, MEH17003, GOP17001.2, RAJ17001, CHU16001.3 and JES16001, that had shown high pathogenicity and high capacity of sporulation. Local varieties BARI Gom 26 and BARI Gom 33 were used as the susceptible and resistant check, respectively. The nurseries were surrounded by spreader rows of susceptible line BARI Gom 26, that were inoculated with MoT isolates 4–5 times between the seedling to the heading stage.

A misting system was set up in each nursery, operating from 8 a.m. to 7 p.m. in Bolivia and 9 a.m. to 5 p.m. in Bangladesh during the period of disease development, with 10–15 min per hour of water misting to keep a humid micro-environment that is conducive for wheat blast development. Blast evaluations were done 20–22 days after the first inoculation, when the susceptible checks had greater than 80% blast severity. The total and infected number of spikelets on ten spikes that were tagged at anthesis were then recorded. Blast incidence was calculated as the proportion of spikes with blast infection and the blast severity was calculated as the average percentage of spikelets that were infected. The incidence and severity values were then used to estimate the blast index using the formula: wheat blast index = incidence × severity. We also calculated a mean blast index across all the environments for each nursery. The blast indices for all the lines are given in Supplementary Table S1 and we also obtained the mean, median, minimum, range, standard deviation, standard error of the mean and variance of the blast indices. In addition, we obtained the Pearson’s correlation between the blast indices in the different environments and the narrow-sense heritabilities across the planting times in each environment using the formula:

$$h^{2} = \frac{{\sigma_{g}^{2} }}{{\sigma_{g}^{2} + \frac{{\sigma_{\varepsilon }^{2} }}{nplantings}}},$$

where $\sigma_{g}^{2}$ was the genetic variance among the lines calculated using the markers, $\sigma_{\varepsilon }^{2}$ is the error variance, and *nplantings* is the number of planting dates. We estimated the genetic and residual variances using the average information-restricted maximum likelihood algorithm^62^ in the ‘R’ package ‘heritability’^63^.

### Genotyping data

All the 1106 lines in the four nurseries were genotyped using the genotyping-by-sequencing method^64^ and the marker polymorphisms were called using the TASSEL (Trait Analysis by aSSociation Evolution and Linkage) version 5 GBS pipeline^65^. Marker polymorphisms were discovered using a minor allele frequency of 0.01, that resulted in 6,075,743 tags, among which 64% aligned to the reference genome (RefSeq v1.0) assembly of Chinese Spring^66^. After filtering the tags as described in Juliana et al., we obtained 78,662 single-nucleotide polymorphisms. We then filtered the markers in each IBWSN and SAWSN for missing data less than 60%, minor allele frequency greater than 10% and heterozygosity less than 10%, resulting in 8053 markers in the 50 IBWSN, 8126 markers in the 35 SAWSN, 7555 markers in the 51 IBWSN and 7795 markers in the 36 SAWSN. Similarly, the combined panel with all the IBWSNs and SAWSNs was also filtered for missing data less than 60%, minor allele frequency greater than 5% and heterozygosity less than 10%, resulting in 8072 markers . The lines in the different nurseries were also filtered for missing data resulting in 263 lines in the 50 IBWSN, 255 lines in the 35 SAWSN, 271 lines in the 51 IBWSN, 268 lines in the 36 SAWSN and 1048 lines in the combined panel with good genotyping data. The linkage disequilibrium k-nearest neighbor genotype imputation method^67^ was used for imputation in TASSEL^68^.

### Genome‑wide association mapping for blast resistance

We performed genome-wide association studies for blast resistance in 49 datasets including: (i) 18 datasets with the 50 IBWSN and 35 SAWSN evaluations in the first and second plantings at Jashore 2019, Quirusillas 2019, Okinawa 2018, Quirusillas 2018 and the mean across environments (ii) 18 datasets with the 51 IBWSN and 36 SAWSN evaluations in the first and second plantings at Jashore, Okinawa and Quirusillas 2019, Quirusillas 2020 and the mean across these environments (iii) 13 datasets with the combined panel evaluations in the first and second plantings at Jashore 2019, Okinawa 2018 and 2019, Quirusillas 2018, 2019 and 2020 and the mean across these environments.

Marker-trait association tests were done in TASSEL version 5 using the mixed linear model^69^ with the optimum level of compression and the ‘population parameters previously determined’ option^70^. The first two principal components accounting for the population structure^71^ were used as fixed effects and the kinship matrix among individuals estimated using the centered identity-by-state method^72^ was used as the covariance matrix for the random genotypic effect in the mixed linear model. The principal components were then used to cluster the lines into five arbitrary clusters based on the k-means clustering approach in the Jmp statistical software (www.jmp.com). We also estimated the individual ancestry coefficients of the lines and the number of ancestral sub-populations using the ‘R’ package LEA (Landscape and Ecological Association studies) based on the value of the cross-entropy criterion^73^. The p-values, additive effects and percentage variation explained by each marker were obtained and Manhattan plots with the − log_10_ p-values of the markers were plotted using the ‘R’ package CMplot^74^. The Bonferroni method for multiple testing correction at an α level of 0.20 was used to declare significance of the markers. The markers that were consistently associated with blast resistance in more than one dataset were identified and their genetic positions in the Popseq map^75^ were obtained.

### Genomic fingerprinting and frequencies of favorable alleles

The effects of the consistent markers significant in more than one dataset estimated from the mixed linear model were analyzed and the alleles that had a decreasing effect on the blast index (favorable alleles, FAs), an increasing effect on the blast index (unfavorable allele) and the heterozygote were identified. These alleles were then used to fingerprint the 1106 lines in all the nurseries to understand the allelic composition of lines for blast resistance associated markers. A heatmap with the fingerprinted markers was obtained with the ‘R’ package ‘pheatmap’^76^. We also plotted the marker alleles in the 1106 lines with and without the 2NS translocation against their mean blast indices in Jashore 2019, Okinawa 2018, Quirusillas 2018 and Quirusillas 2019 using the ‘R’ package ggplot2^77^.

### Frequencies of lines with the 2NS translocation in the IBWSNs and SAWSNs

To validate whether the most consistent regions identified in this study referred to the 2NS translocation, we used the Kompetitive allele specific polymerase chain reaction co-dominant marker CIMwMAS0004 that is specific for the translocation^50–52^ and compared the presence of the translocation with the FAs at the most significant markers on chromosome 2AS identified in this study. After validating the association of the most significant markers on chromosome 2AS to the 2NS translocation, we estimated the frequencies of lines with FAs at the 2NS translocation across all the IBWSNs and SAWSNs distributed internationally between 2012 and 2019 using 4143 lines from eight IBWSNs and SAWSNs. This included the following number of genotyped lines in sixteen nurseries indicated by the year of their distribution and the nursery name: (i) 2012 45 IBWSN—323 lines (ii) 2013 46 IBWSN—301 lines (iii) 2014 47 IBWSN—285 lines (iv) 2015 48 IBWSN—283 lines (v) 2016 49 IBWSN—275 lines (vi) 2017 50 IBWSN—267 lines (vii) 2018 51 IBWSN—284 lines (viii) 2019 52 IBWSN—266 lines (ix) 2012 30 SAWSN—96 lines (x) 2013 31 SAWSN—204 lines (xi) 2014 32 SAWSN—194 lines (xii) 2015 33 SAWSN—270 lines (xiii) 2016 34 SAWSN—257 lines (xiv) 2017 35 SAWSN—261 lines (xv) 2018 36 SAWSN—285 lines (xvi) 2019 37 SAWSN—264 lines.

## Supplementary information

Supplementary Figure 1.

Supplementary Figure 2.

Supplementary Figure 3.

Supplementary Figure 4.

Supplementary Figure 5.

Supplementary Figure 6.

Supplementary Legends.

Supplementary Information 1.

Supplementary Information 2.

## Data Availability

The blast phenotyping data of 1106 lines evaluated in different environments is available in Supplementary Table S1.
