# Supplementary figures and images for: Genome-wide association mapping for wheat blast resistance in CIMMYT’s international screening nurseries evaluated in Bolivia and Bangladesh
Source: Sci Rep. 2020 Oct 2;10:15972. doi: 10.1038/s41598-020-72735-8 (PMC7532450; doi:10.1038/s41598-020-72735-8)

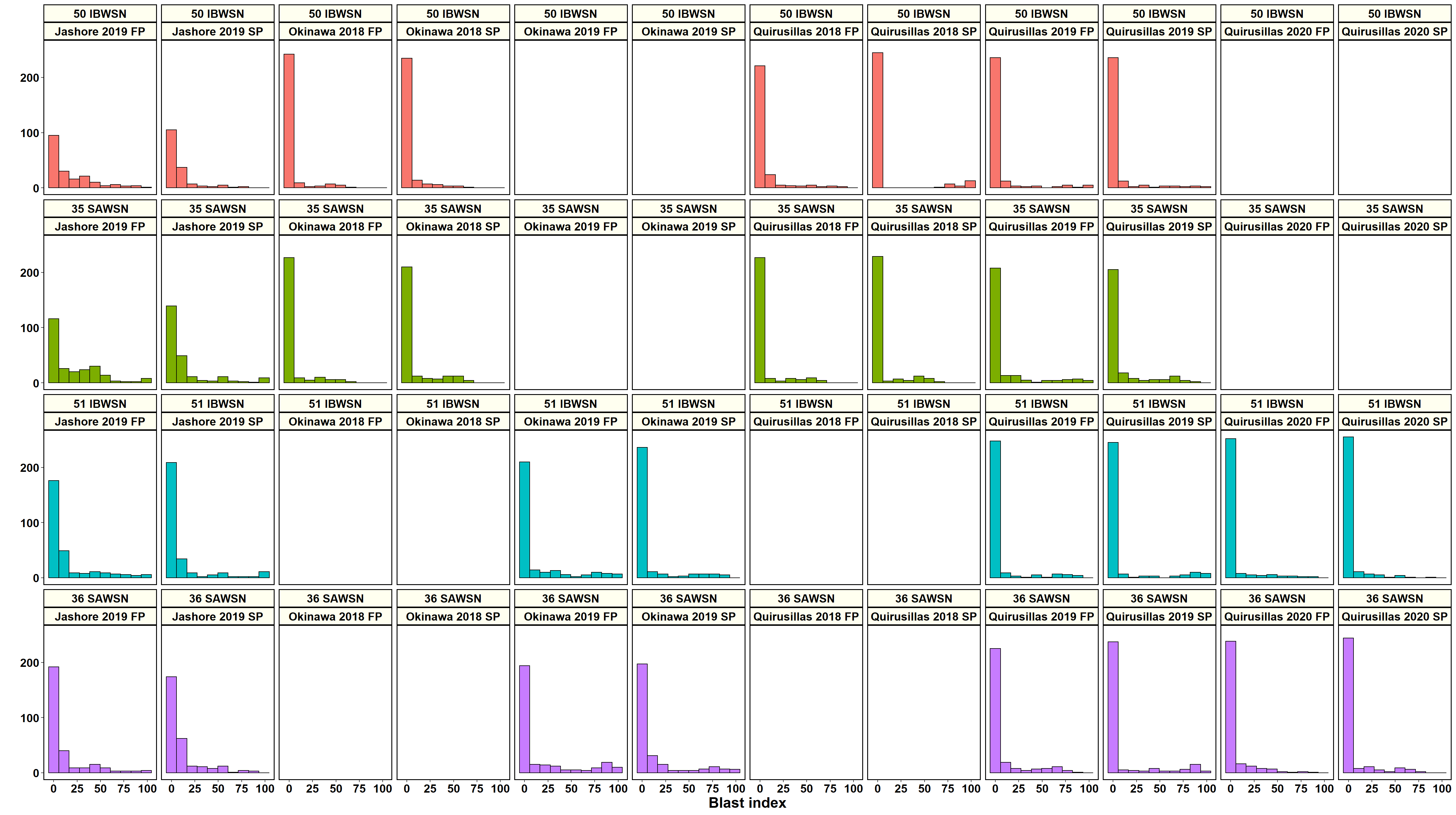

Supplement: Supplementary file 1 — Supplementary Figure 1. [file 41598_2020_72735_MOESM1_ESM.tiff]

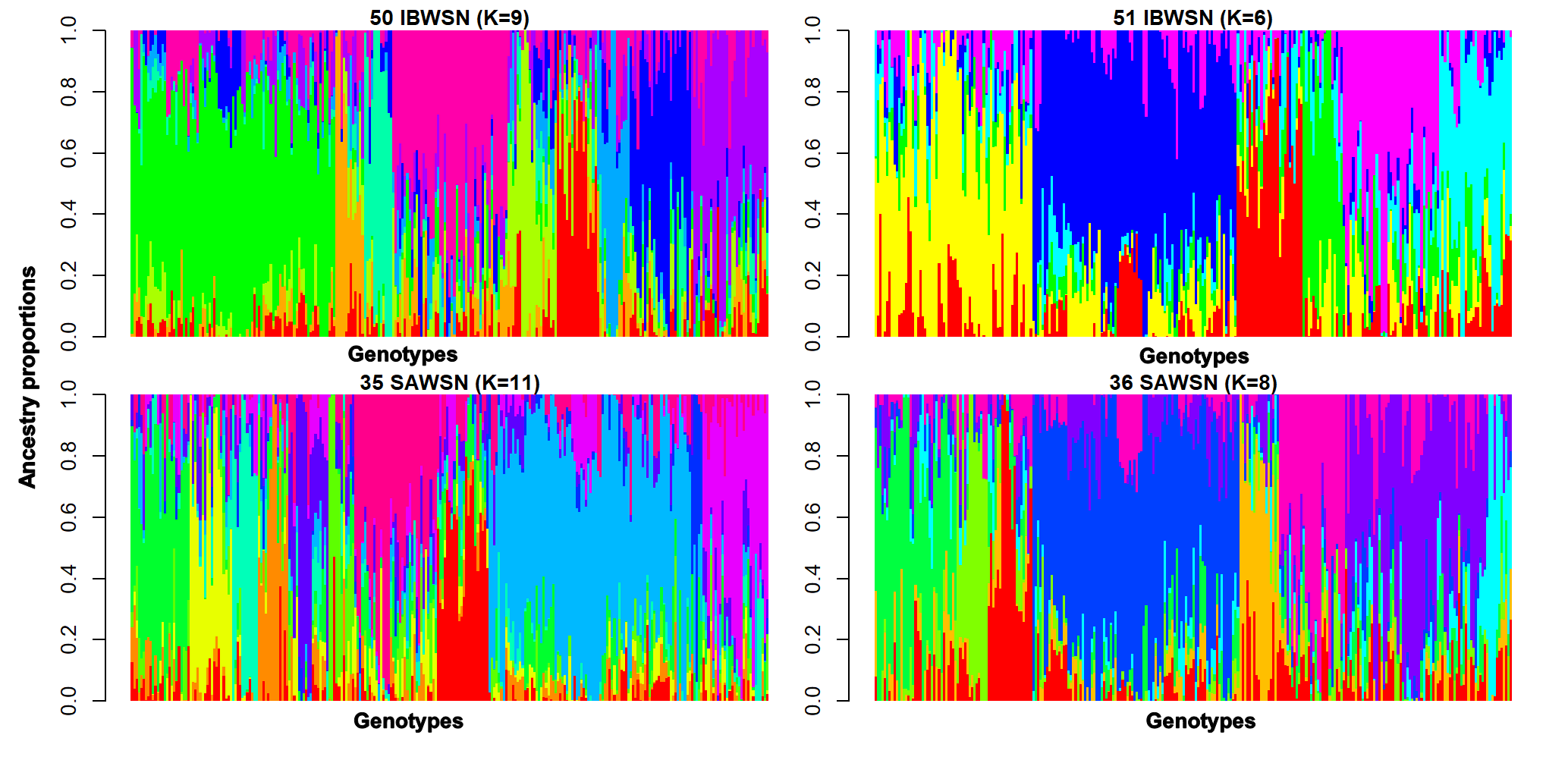

Supplement: Supplementary file 2 — Supplementary Figure 2. [file 41598_2020_72735_MOESM2_ESM.tiff]

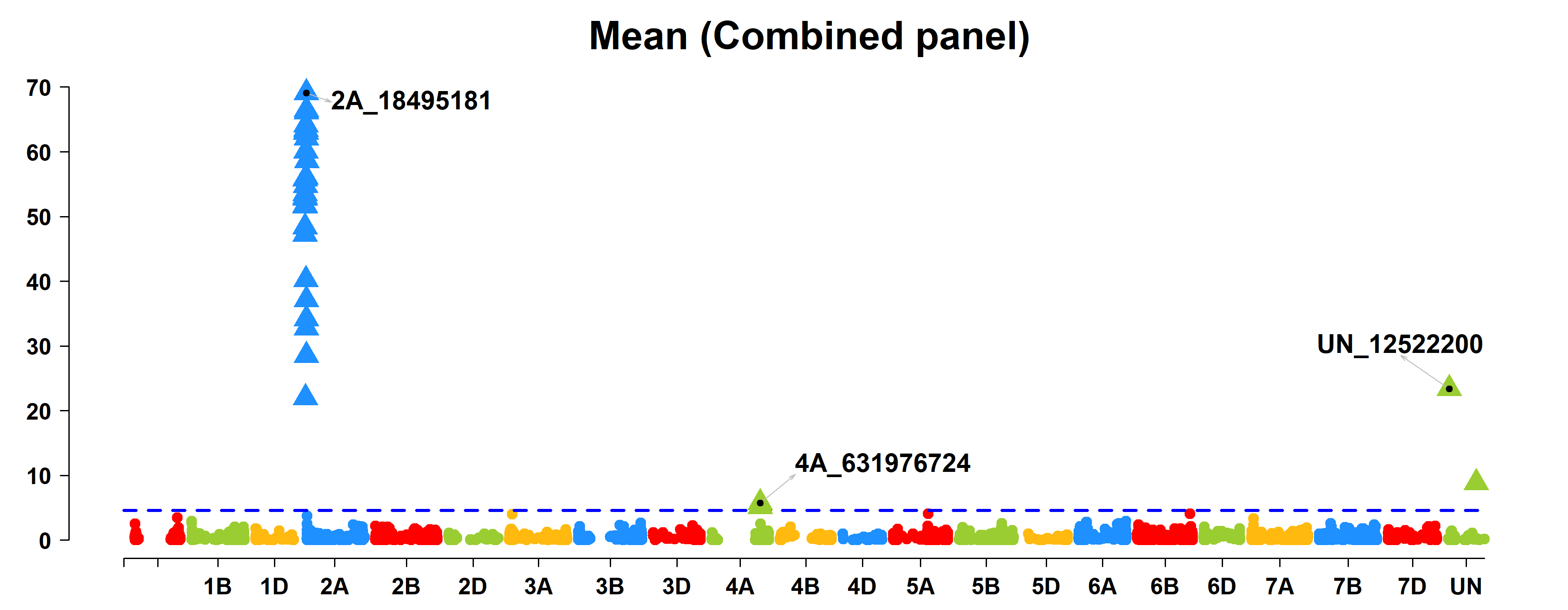

Supplement: Supplementary file 3 — Supplementary Figure 3. [file 41598_2020_72735_MOESM3_ESM.tiff]

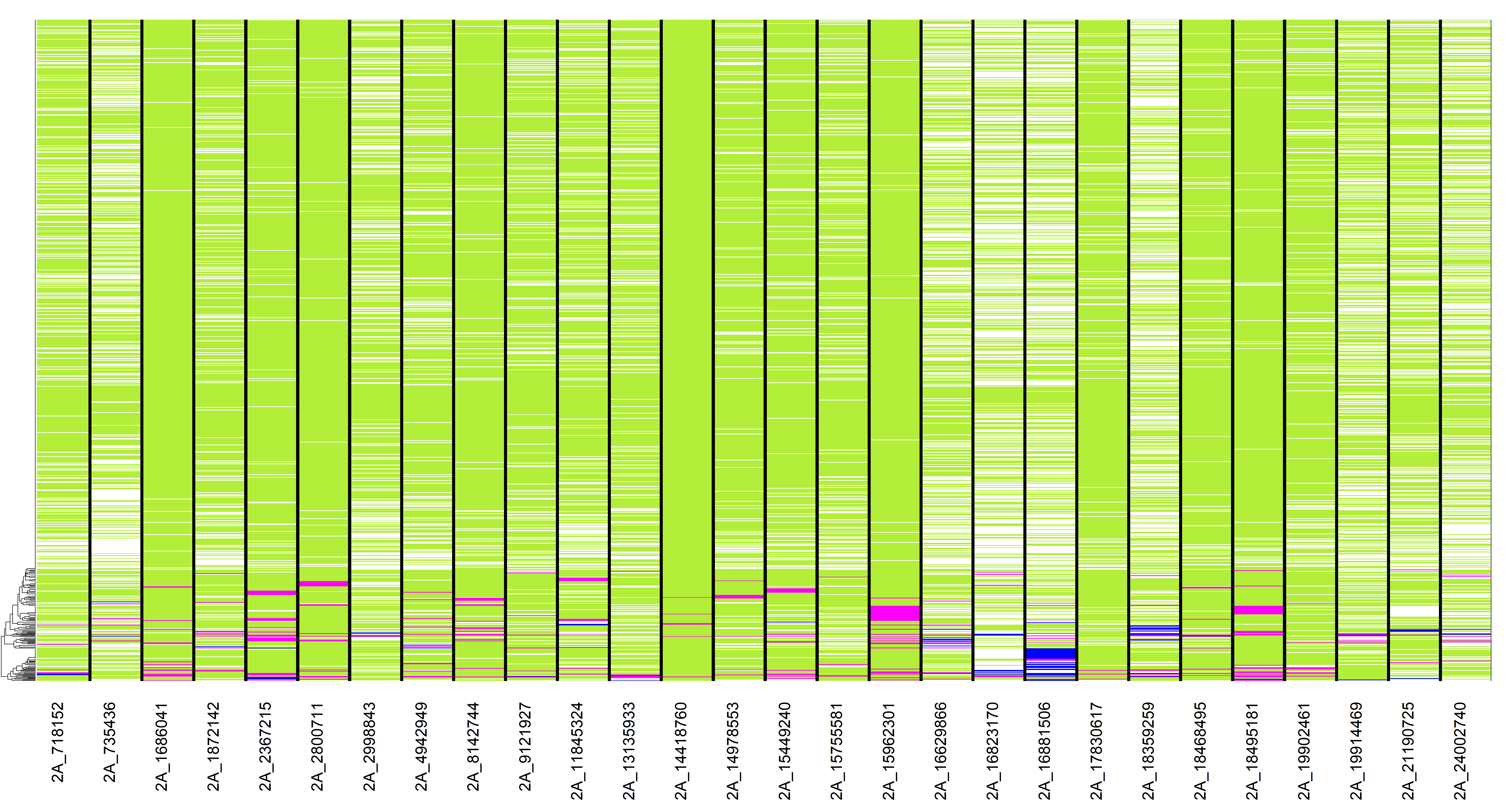

Supplement: Supplementary file 4 — Supplementary Figure 4. [file 41598_2020_72735_MOESM4_ESM.tiff]

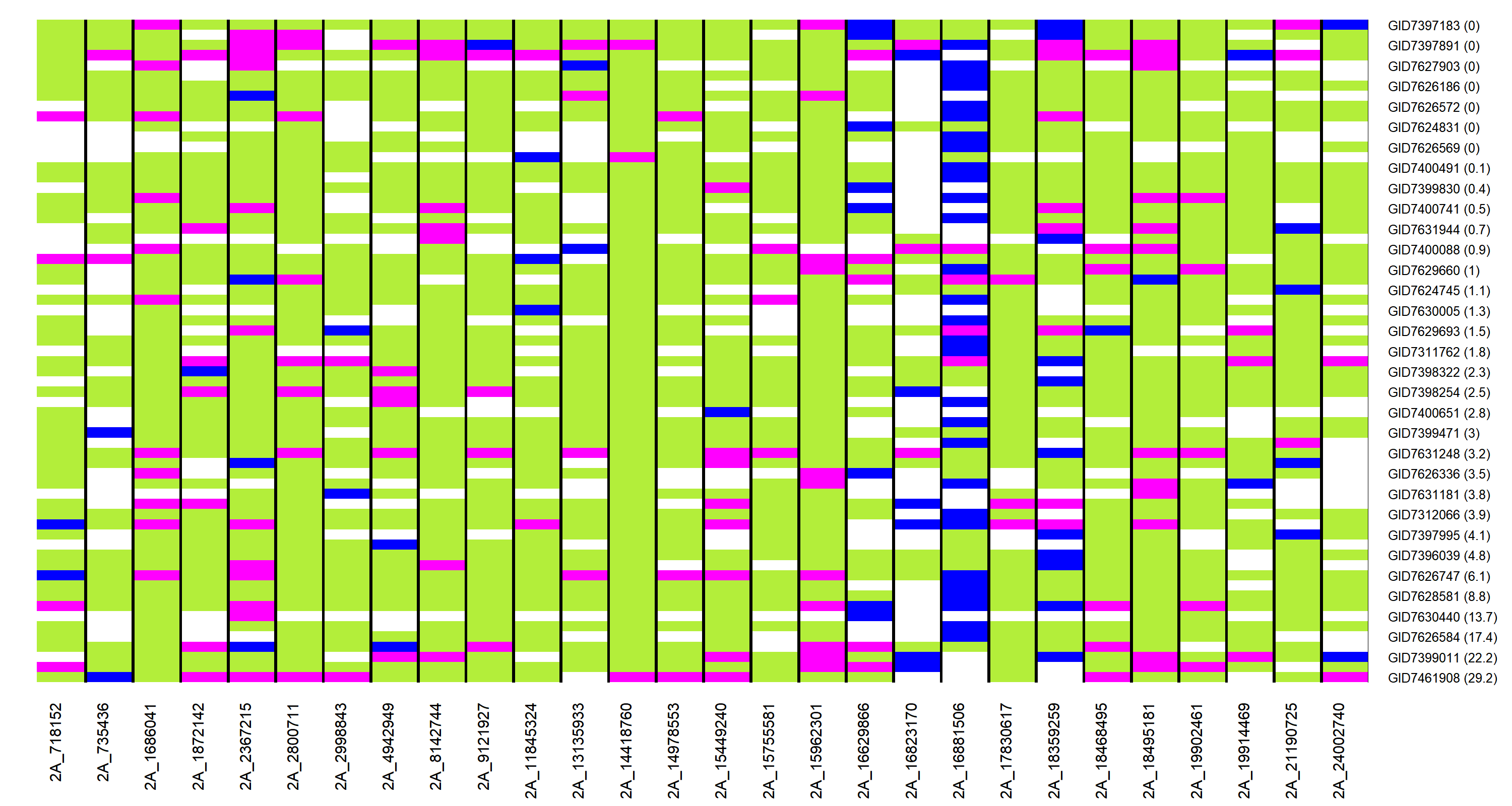

Supplement: Supplementary file 5 — Supplementary Figure 5. [file 41598_2020_72735_MOESM5_ESM.tiff]

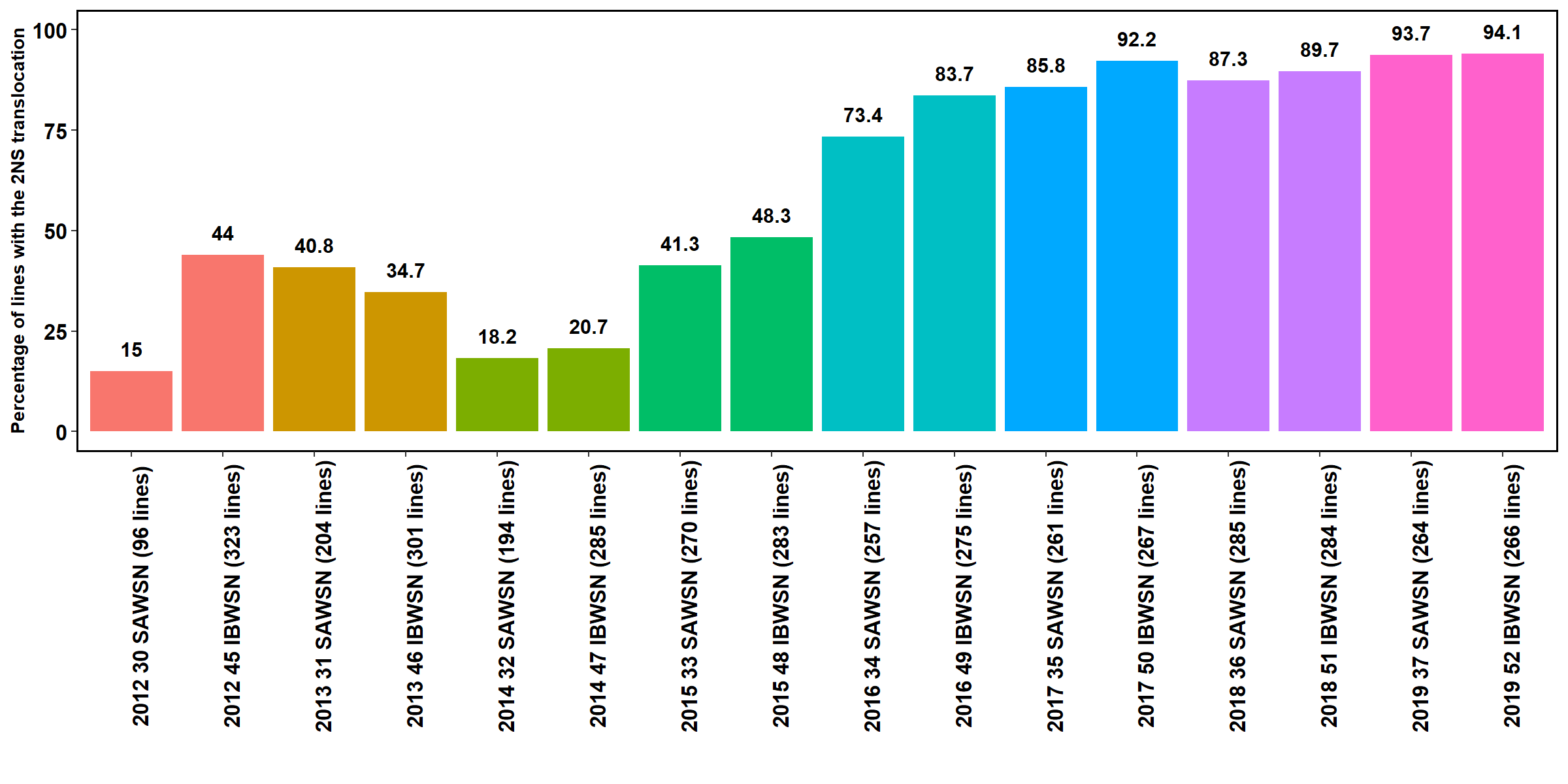

Supplement: Supplementary file 6 — Supplementary Figure 6. [file 41598_2020_72735_MOESM6_ESM.tiff]
