# Supplementary material for: Genome-wide association mapping for wheat blast resistance in CIMMYT’s international screening nurseries evaluated in Bolivia and Bangladesh
Source: Sci Rep. 2020 Oct 2;10:15972. doi: 10.1038/s41598-020-72735-8 (PMC7532450; doi:10.1038/s41598-020-72735-8)
Supplement: Supplementary file 7 — Supplementary Legends. [file 41598_2020_72735_MOESM7_ESM.docx]

**Figure S1:** Distribution of blast indices in the 50 and 51 International Bread Wheat Screening nurseries (IBWSN) and 35 and 36 Semi-Arid Wheat Screening Nurseries (SAWSN) in the first planting (FP) and second planting (SP) at the Jashore 2019, Okinawa 2018, Okinawa 2019, Quirusillas 2018, Quirusillas 2019 and Quirusillas 2020 environments.

**Figure S2:** Population structure analysis of the lines in the 50 and 51 International Bread Wheat Screening nurseries (IBWSN) and 35 and 36 Semi-Arid Wheat Screening Nurseries (SAWSN) showing the ancestry proportions and the ancestral sub-populations obtained using the ‘R’ package LEA (Landscape and Ecological Association Studies).

**Figure S3:** Markers significantly associated with mean blast indices in the combined panel comprising the 50 and 51 International Bread Wheat Screening nurseries, 35 and 36 Semi-Arid Wheat Screening Nurseries. A Bonferroni α level of 0.20 was used to correct for multiple testing and the most significant marker in each chromosome is indicated.

**Figure S4:** Clustering of 915 lines from the 50 and 51 International Bread Wheat Screening nurseries and 35 and 36 Semi-Arid Wheat Screening Nurseries based on the favorable alleles at 28 blast associated markers in the 2NS translocation. The green color indicates the favorable allele (allele with a decreasing effect on the blast index), the blue color indicates the unfavorable allele (allele with an increasing effect on the blast index), the magenta color indicates the heterozygote and the white color indicates missing data.

**Figure S5:** The genomic fingerprints of 65 lines with a high number of favorable alleles at the 2NS translocation in addition to some unfavorable alleles and heterozygotes, sorted based on their blast indices given in parenthesis after the genotype’s identifier. The green color indicates the favorable allele (allele with a decreasing effect on the blast index), the blue color indicates the unfavorable allele (allele with an increasing effect on the blast index), the magenta color indicates the heterozygote and the white color indicates missing data.

### Figure S6: Percentage of lines with favorable alleles for blast in the 2NS translocation. The percentage of lines with favorable alleles were obtained in eight International Bread Wheat Screening nurseries (45-52 IBWSN) and eight Semi-Arid Wheat Screening Nurseries (30-37 SAWSN) that were distributed internationally between 2012 and 2019.
